# Relationship Between the Serum Cortisol, Insulin, Adrenocorticotropic Hormone (ACTH), and Blood Glucose Levels of Pregnant Women With Gestational Diabetes Mellitus in the Kingdom of Bahrain

**DOI:** 10.7759/cureus.71782

**Published:** 2024-10-18

**Authors:** Tarik AlShaibani, Wadeea Gherbal, Amer Almarabheh, Diaa Rizk, Moudhi Esmaeel, Reem Alhouli, Nora AlGhareeb, Hajar Alenezi, Sharifa Alzayani, Husain Taha, Amal A Hassani, Yahya Naguib

**Affiliations:** 1 Physiology Department, College of Medicine and Health Sciences, Arabian Gulf University, Manama, BHR; 2 Obstetrics and Gynecology Department, Salmaniya Medical Complex, Manama, BHR; 3 Family and Community Medicine Department, College of Medicine and Health Sciences, Arabian Gulf University, Manama, BHR; 4 Obstetrics and Gynaecology Department, College of Medicine and Health Sciences, Arabian Gulf University, Manama, BHR; 5 Internal Medicine Department, Endocrine Unit, Salmaniya Medical Complex, Manama, BHR; 6 Clinical Physiology Department, Faculty of Medicine, Menoufia University, Shebin El Kom, EGY

**Keywords:** cortisol, gestational diabetes mellitus, glucose, insulin, pregnancy, stress, stress hormones.

## Abstract

Background

Gestational diabetes mellitus (GDM) can be defined as hyperglycemia that develops during pregnancy. GDM poses both maternal and fetal potential risks. Elevated maternal cortisol levels have been linked to maternal hyperglycemia and insulin resistance. The present study aimed to investigate the relationship between GDM and serum cortisol levels in Bahraini and non-Bahraini pregnant women in the Kingdom of Bahrain. We also investigated the relationship between age and ethnicity in the development of GDM.

Methods

Data were collected from a total of 75 pregnant women; 41 of which were diagnosed with GDM and 34 had normal blood glucose levels. Serum cortisol, insulin, adrenocorticotropic hormone (ACTH), fasting (FBG), and random (RBG) blood glucose levels were measured.

Results

FBG, RBG, and insulin blood levels were significantly higher in the GDM group when compared to the control group. Serum cortisol and ACTH levels tended to be higher in the GDM group; however, they were statistically insignificant. Within the GDM group, there were no statistically significant differences in serum insulin, cortisol, and ACTH levels between Bahraini and non-Bahraini patients or between patients less than or more than 30 years old.

Conclusion

Our results suggest that cortisol may not have a major role in the development of GDM in our patients. Further research is needed to confirm these results. This study highlights the necessity to better understand the underlying mechanisms of the development of GDM in pregnant women.

## Introduction

Gestational diabetes mellitus (GDM) is defined as high blood glucose levels that develop during pregnancy and is usually diagnosed at 24 to 28 weeks of gestation based on elevated plasma glucose levels on glucose tolerance testing. GDM is generally a state of both enhanced beta-cell function and marked insulin resistance mediated primarily by placental secretion of diabetogenic hormones, including human placental lactogen, growth hormone, prolactin, corticotropin-releasing hormone (CRH), and progesterone. These and other metabolic changes are most prominent in the third trimester, primarily to ensure that the fetus has a sufficient supply of nutrients [1].

Normally, insulin is secreted from the pancreas to lower the blood glucose level by stimulating glucose deposition in cells [2]. It is common and completely normal for women to go through very arduous and stressful conditions during pregnancy. However, too much stress can be overwhelming and could even lead to health problems. During times of stress, the body releases cortisol to help itself respond to stress and provide it with glucose by tapping into protein stores via gluconeogenesis in the liver [3]. However, consistent and prolonged elevation in cortisol levels contributes to the development of GDM, which can lead to both maternal and fetal health problems [4]. In fact, several observational studies found an association between elevated serum cortisol levels and GDM [5]. Additionally, animal studies demonstrated that glucocorticoids can induce insulin resistance in skeletal muscles resulting in a decrease in glucose utilization and an increase in fat consumption for energy supply [6].

Cortisol is an essential hormone that moderates the stress response. Cortisol is a steroid hormone that can be synthesized from cholesterol in the zona fasciculata of the adrenal cortex. Cortisol secretion is under the control of the hypothalamus-pituitary-adrenal (HPA) axis. Glucocorticoid receptors are present in almost all tissues in the body, and thereby, cortisol acts on almost every organ system. The human body is continually responding to internal and external stressors. The body processes stressful information and elicits a response depending on the degree of threat [7]. Cortisol affects glucose homeostasis in several aspects. In skeletal muscles, cortisol reduces the translocation of the insulin-dependent glucose transporter 4 (GLUT4) to the plasma membrane, resulting in the impairment of glucose uptake. Simultaneously, cortisol activates glycogen synthase kinase-3, which suppresses glycogen synthesis and promotes protein degradation [8]. In adipose tissues, cortisol increases lipolysis. The abundance of free fatty acids (FFAs) can be used in β-oxidation as an energy source for other cells [7]. Lastly, cortisol is a potent insulin antagonist, which is capable of inhibiting insulin secretion, stimulating glucagon secretion, and disrupting insulin signaling [9].

GDM is thought to be a result of a combination of hormonal changes during pregnancy [10]. The underlying mechanism of the development of GDM remains unclear. Elevated levels of serum cortisol in pregnant women can contribute to the pathogenesis of GDM. The body remains in a general insulin-resistant state when cortisol levels are chronically elevated. Over time, a vicious cycle develops in which the pancreas struggles to maintain insulin production, the glucose levels rise in the blood, and the cells fail to utilize blood glucose [4]. Interestingly, studies found that apart from physiological factors, anxiety and depression are also important risk factors for GDM [11]. Antenatal anxiety and depression may result in chronic HPA hyperactivity, resulting in enhanced release of cortisol with subsequent development of insulin resistance and increased risk of developing GDM [12]. The incidence of GDM has been shown to be higher in pregnant women with anxiety symptoms [13].

We previously reported that insulin levels were higher in pregnant women with GDM irrespective of their ethnicity or age. We concluded that the disrupted control of blood glucose in GDM despite the presence of high insulin secretion may suggest a loss of insulin effectiveness due to other factors, including stress [14]. Herein, we studied the relationship between GDM and serum cortisol levels in pregnant women in the Kingdom of Bahrain based on both ethnicity and age. This study also aimed to investigate the relationship between insulin levels and HPA activity, which could be concluded by cortisol and adrenocorticotropic hormone (ACTH) serum levels in those pregnant women.

## Materials and methods

Ethical considerations

All procedures were in accordance with the Declaration of Helsinki and the guidelines of the Research and Ethics Committee (REC) at the College of Medicine and Health Sciences, Arabian Gulf University, Kingdom of Bahrain. Ethical approval number E1-P1-10-22 was granted by REC in October 2021. Before obtaining informed consent, all participants were fully informed about the study and were given the right to quit at any time they wished. Participants were given a copy of their test results.

Study design and data collection

Data were collected from the Obstetrics and Gynecology Clinics at Salmaniya Medical Complex, Manama, Kingdom of Bahrain, between January 2022 and Jan 2024. Blood samples were collected from two groups: pregnant women with GDM (GDM group) and non-diabetic pregnant women (Control group). Blood glucose, cortisol, ACTH, and insulin levels were measured using a reliable and standardized assay method.

The total number of pregnant women was 75, and they were between 23 weeks to full term of gestation. Pregnant women were recruited from the inpatient and outpatient clinics. Participants were then classified into the GDM group (number = 41) and the Control group (number = 34). Sociodemographic data were initially collected. Following that, multiple blood samples were collected as required.

First, random blood samples were collected to measure RBG levels. Then, participants were asked to fast for six to eight hours overnight, and fasting blood samples were collected early the next morning. FBG, insulin, cortisol, and ACTH levels were then measured using the Atellica Solution Immunoassay and Clinical Chemistry Analyzers (Siemens Healthineers AG, Forchheim, Germany).

Inclusion criteria

Pregnant women with GDM are diagnosed based on the established diagnostic criteria (abnormal glucose tolerance test). Non-diabetic pregnant women with no history of diabetes or any signs/symptoms suggestive of GDM were included as controls. The age range was set between 18 and 45 years.

Exclusion criteria

Pregnant women with pre-existing type I or type II diabetes, pregnant women with pre-existing hypertension, renal disease, or autoimmune disorders, and pregnant women on insulin-influencing medications. Pregnant women above the age of 45 years were excluded from the study.

Data management and statistical analysis plan

Data were analyzed as described before [14]. Briefly, we performed the Shapiro-Wilk normality test to assess the normal distribution of all clinical and biological parameters. Categorical variables were signified as frequencies and percentages while continuous variables were presented as mean and standard deviation. Independent two-sample student’s t-test was used to test the significance between the GDM and control groups. Pearson's correlation coefficient (r) was used to determine the relationships between parameters. Data were analyzed using the Statistical Package for Social Sciences (SPSS), version 29 (IBM Corp., Armonk, NY, USA). A p-value < 0.05 was considered statistically significant [15,16].

## Results

The results demonstrated higher blood levels of FBG, RBG, insulin, cortisol, and ACTH in the GDM group when compared to the control group. The results of the independent samples t-test indicated that there were statistically significant differences between the two groups in terms of FBG (t=2.265, p=0.026), RBG (t=2.836, p=0.006), and insulin levels (t=2.320, p=0.023), with high effect size (Cohen’s d=0.525, 0.658, 0.567), respectively. Regarding cortisol and ACTH, the results revealed insignificant differences between the two groups (P>0.05) (Table 1).

**Table 1:** Comparison of the tested biomarkers of glucose homeostasis and stress hormones between the GDM and control groups Independent samples t-test, * significant (P<0.05) GDM: gestational diabetes mellitus, FBG: fasting blood glucose, RBG: random blood glucose, ACTH: adrenocorticotropic hormone

Factors	Group				Statistics (T-value)	P-Value	Effect size
	GDM (n=41)		Test (n=34)				
	Mean	St. Error	Mean	St. Error			
FBG	5.40	0.22	4.71	0.20	2.265	0.026*	0.525
RBG	6.30	0.33	5.15	0.19	2.836	0.006*	0.658
Insulin	77.01	18.24	30.32	6.67	2.320	0.023*	0.567
Cortisol	663.20	34.57	641.33	32.12	0.454	0.651	0.106
ACTH	10.13	1.14	9.19	0.97	0.617	0.539	0.145

**Figure 1:**
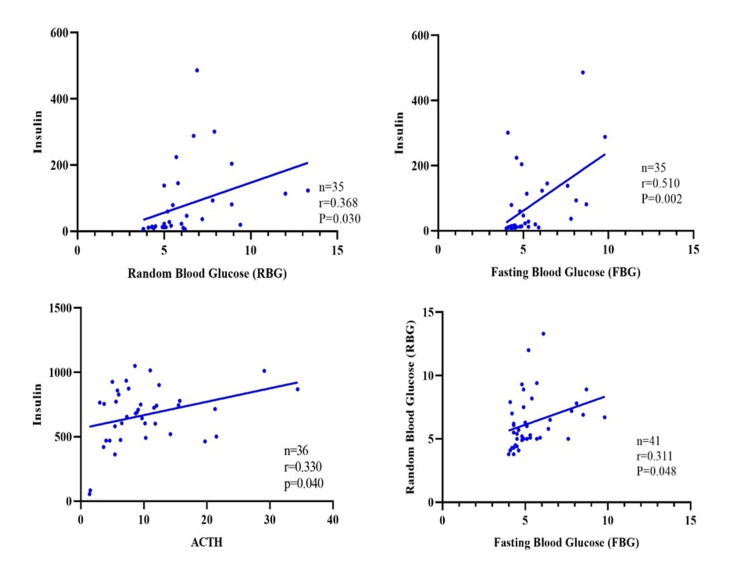
The relationship between the tested biomarker for glucose homeostasis and stress hormones in the GDM group (r: Pearson correlation coefficient) GDM: gestational diabetes mellitus

The results for the GDM group revealed a positive relationship between FBG and RBG (correlation coefficient (r)=0.311, sample size (n)=41, p-value (p)=0.048), which indicated that they tend to rise or fall together. Regarding insulin, the results showed that there was a positive relationship between insulin and both FBG and RBG (r=0.510, n=35, p=0.002 and r=0.368, n=35, and p=0.030, respectively), indicating a relationship where higher levels of insulin were associated with higher levels of both FBG and RBG levels. Results also showed that there was a positive relationship between insulin and ACTH (r=0.330, n=36, p=0.040). Taken together, the findings supported the presence of significant positive relationships between these biomarkers, highlighting potential coregulation and/or mutual influences in the context of glucose homeostasis in pregnant women in the Kingdom of Bahrain (Figure 1). It has to be noted that when assessing the value of the Pearson correlation coefficient between two quantitative (continuous) variables, missing values are excluded from the analysis for a given sample of patients, even if they are present for one variable but not the other. As a result, the number of observations must be consistent for both variables to calculate a reliable correlation coefficient. Consequently, variations in sample size occur when examining the correlation between different pairs of variables due to this requirement for data consistency in correlation analyses.

The results revealed that there were no statistically significant differences between Bahraini and non-Bahraini patients in the GDM group regarding insulin, cortisol, and ACTH levels (p=0.072, p=0.222, and p=0.613), respectively (Table 2).

**Table 2:** Comparison of insulin, cortisol, and ACTH levels for GDM group according to the ethnicity of the patients GDM: gestational diabetes mellitus, FBG: fasting blood glucose, RBG: random blood glucose, ACTH: adrenocorticotropic hormone

Factors	Nationality				P. Value
	Bahraini (n=12)		Non-Bahraini (n=29)		
	Mean	St. Error	Mean	St. Error	
Insulin	25.23	8.08	97.73	24.24	0.072
Cortisol	729.42	78.69	635.80	36.27	0.222
ACTH	9.25	1.44	10.52	1.53	0.613

The results indicated that there were no statistically significant differences between patients whose ages were less than 30 years old and those who were ages 30 years and above in the GDM group in terms of insulin, cortisol, and ACTH blood levels (p=0.133, p=0.706, and p=0.670) respectively (Table 3).

**Table 3:** Comparison of insulin, cortisol, and ACTH levels for GDM group according to the age of the patients GDM: gestational diabetes mellitus, FBG: fasting blood glucose, RBG: random blood glucose, ACTH: adrenocorticotropic hormone

Factors	Age group				P-value
	< 30 Years (n=13)		≥ 30 Years (n=28)		
	Mean	St. Error	Mean	St. Error	
Insulin	38.88	14.22	96.91	26.03	0.133
Cortisol	643.69	73.09	672.25	38.48	0.706
ACTH	9.43	1.66	10.48	1.51	0.670

## Discussion

Cortisol, the primary stress hormone, has the opposite effect to insulin, as it increases the level of blood glucose. Pregnancy is a very stressful state or condition for any pregnant woman, which, presumably, may lead to more secretion of cortisol and, in turn, will increase the glucose level in the bloodstream of the pregnant woman. The purpose of this study was to detect if there is a relationship between GDM and serum cortisol levels in pregnant women in the Kingdom of Bahrain. Additionally, we investigated the possibility of having ethnic or age differences in pregnant women with GDM. The relationship between insulin and HPA activity was also studied.

The present study showed that pregnant women with GDM had significantly higher insulin levels compared to their counterparts. No significant differences in serum cortisol or ACTH levels could be detected between pregnant women with or without GDM. In line with our findings, a case-control study of 96 pregnant females found a significantly higher level of insulin among pregnant women with GDM [17]. On the contrary, other studies reported no change in insulin levels among pregnant women with or without GDM [18].

In the present study, serum cortisol levels were insignificantly higher in pregnant women with GDM when compared with their counterparts without GDM. There is a clear variation in the association between cortisol levels and GDM in the literature. However, it is quite accepted that higher levels of stress hormones, including cortisol, have a role in the pathogenesis of GDM [19]. Cortisol is a potent insulin antagonist that inhibits insulin secretion, stimulates glucagon secretion, and disrupts insulin signaling [20].

Previously published data were inconsistent regarding cortisol levels and GDM. Several studies reported higher levels of cortisol in pregnant women with GDM when compared to their counterparts of pregnant women with normal blood glucose levels. In addition, they even correlated the severity of GDM and the glycemic parameters with serum cortisol levels. A previously published cross-sectional study reported a correlation between maternal blood cortisol and glucose levels [4]. In contrast, other studies reported low levels of serum cortisol among pregnant women with GDM. A previously published study reported lower cortisol, but higher cortisone, levels among pregnant women with GDM [21]. Shriyan et al., in a prospective cohort study, demonstrated that there was no association between serum cortisol levels and GDM status. Furthermore, they reported that GDM rates were similar regardless of the levels of maternal blood cortisol level [22]. However, in favor of our results, another case-control study reported that serum cortisol levels were insignificantly higher among pregnant women with GDM [23], which is similar to that reported in the present study.

In line with the findings of the present study, many studies revealed that ACTH levels are not associated with maternal GDM status. For instance, a study by Chiodini et al. found that pregnant women with GDM had comparable ACTH levels to those without GDM [24]. Several studies showed variation in the ACTH and cortisol levels and efficacy with age among females; higher levels and efficacy were reported among elderly populations [25,26]. In contrast, some studies reported no differences in cortisol levels across all ages [27].

We did not find any association between sociodemographic characteristics, including patients’ age and ethnicity, and the blood levels of insulin, cortisol, and ACTH. This indicated, at least to us, that both age and ethnicity play no role in the levels of the assessed hormones during pregnancy. In contrast to our findings, some studies reported an association between patients’ ethnicity and plasma stress hormone levels. Cortisol levels were lower among African American pregnant patients while ACTH levels were higher among them when compared to Hispanic pregnant women [28].

Limitations

This study has some limitations. A relatively small sample size was recruited. Only three main hormones were assessed. The assessment of cortisone, glucagon, and CRH among patients with GDM would have given a better insight into the possible pathogenesis of the disease. Additionally, the human placental lactogen and growth hormones should be incorporated in the workup of assessing GDM in Bahraini pregnant women.

## Conclusions

The underlying mechanisms for the development of GDM remain complex and interrelated. Insulin resistance as well as stress hormones play an integral role in the development of GDM. The involvement of cortisol in the development of GDM could vary based on ethnicity; nevertheless, more work should be done to strengthen this assumption.

The present study suggests, at least to us, that HPA activity may not be the main determinant in the development of GDM. This study highlights the necessity to uncover the underlying mechanisms of the development of GDM in pregnant women for better maternal and fetal outcomes.
